# S100A8/A9 as a key player in colorectal cancer: from diagnosis to therapeutic targeting

**DOI:** 10.3389/fphar.2026.1859041

**Published:** 2026-07-17

**Authors:** Yifan Wei, Hua Hao

**Affiliations:** Department of Pathology, Yangpu Hospital, School of Medicine, Tongji University, Shanghai, China

**Keywords:** colorectal cancer, immune regulation, inflammation, S100A8/A9, tumor microenvironment

## Abstract

S100A8/A9, a key member of the calcium-binding protein family, exerts multiple biological effects through its aberrant expression, and serves as an important regulatory factor in the development and progression of colorectal cancer (CRC). Its activity remodels the tumor microenvironment (TME) by amplifying inflammatory responses, establishing immunotolerant conditions, promoting metastatic tumor phenotypes, and forming pre-metastatic and angiogenic niches. These processes suppress anti-tumor immunity, enhance tumor invasion and metastasis, and are associated with poorer prognosis, particularly in advanced-stage CRC. Despite the complexity of its regulatory network, S100A8/A9 represents a valuable potential therapeutic target. This review systematically summarizes the biological properties, clinical significance, and tumor microenvironment-related mechanisms of S100A8/A9 in CRC, with emphasis on inflammation, immune regulation, invasion, metastasis, biomarker potential, and therapeutic targeting. We further discuss emerging S100A8/A9-based intervention strategies, including direct molecular targeting, receptor-pathway blockade, natural-product modulation, combination therapy, and precision-delivery approaches, aiming to clarify both the translational opportunities and limitations of S100A8/A9-directed precision management in CRC.

## 1 Introduction

Colorectal cancer (CRC) is one of the most prevalent malignancies worldwide, with both incidence and mortality rates ranking among the highest (Matsuda et al., 2025; Zhou et al., 2025). According to GLOBOCAN 2022 data, CRC ranks third in global cancer incidence and second in mortality, imposing a substantial disease burden across major countries (Morgan et al., 2023). The 5-year survival rate of CRC patients is strongly associated with disease stage, showing marked prognostic differences: data from the United States (2014–2020) demonstrate that patients with early-stage (stage I-II) CRC achieve 5-year relative survival rates exceeding 90%, whereas those with advanced disease (stage IV) have rates of merely 10%–15%. Consequently, early screening and accurate diagnosis remain critical for reducing CRC-related mortality (Siegel et al., 2023; 2025).

Current treatment strategies for CRC are based on multimodal approaches, including surgery, radiotherapy, adjuvant chemotherapy, targeted therapy, and immunotherapy (Qi et al., 2025). Chemotherapy regimens incorporating 5-fluorouracil (5-FU), oxaliplatin, and irinotecan remain the cornerstone of treatment; however, drug resistance significantly limits their efficacy (Shaham et al., 2025). Targeted agents such as bevacizumab (anti-VEGF antibody) and cetuximab (anti-EGFR antibody) can improve outcomes in selected patients; however, the efficacy of anti-EGFR therapy in particular depends on molecular features such as RAS/BRAF status, and acquired resistance frequently develops (Li et al., 2024; Singh et al., 2024). In recent years, immune checkpoint inhibitors (ICIs) have offered new therapeutic options for CRC (Yan et al., 2024). Notably, ICIs have shown significant efficacy in patients with DNA mismatch repair deficiency (dMMR)-induced microsatellite instability-high (MSI-H) CRC; however, response rates remain limited in most microsatellite stable (MSS) patients. Consequently, drug resistance continues to present a central challenge in the clinical management of CRC (Taieb et al., 2022; Guven et al., 2024).

Growing evidence indicates that the tumor microenvironment (TME) plays a crucial regulatory role in the development, progression, and drug resistance of CRC. Among the key factors, S100A8 and S100A9, two calcium‐binding proteins of the S100 family, have attracted increasing attention. These proteins are mainly secreted by immune cells such as myeloid-derived suppressor cells (MDSCs) and tumor-associated macrophages (TAMs). S100A8/A9 interacts with receptors including the receptor for advanced glycation end products (RAGE) and Toll-like receptor-4 (TLR4), activating downstream signaling pathways such as NF-κB, STAT3, and mitogen-activated protein kinase (MAPK). Through these mechanisms, S100A8/A9 regulates inflammatory responses, promotes tumor-cell proliferation and invasion, and facilitates immune escape (Chen et al., 2023; Zhou et al., 2023). Growing evidence suggests that S100A8/A9 plays a role not only in the tumorigenesis and progression of CRC, but also in metastasis and chemotherapy resistance, indicating its potential as a diagnostic biomarker and therapeutic target of clinical value in CRC (Deguchi and Maru, 2025).

This review systematically summarizes the biological characteristics, biomarker potential, and tumor-microenvironmental mechanisms of S100A8/A9 in CRC, focusing on inflammation, immune regulation, invasion, and metastasis. We also explore potential S100A8/A9-based targeting strategies and their clinical translation prospects, aiming to provide new insights and directions for overcoming drug resistance in CRC.

## 2 Biological characteristics of S100A8/A9 and their clinical significance in CRC

### 2.1 Overview of the structure and function of S100A8/A9

S100A8 and S100A9, also known as myeloid-related proteins 8 and 14 (MRP8 and MRP14), are low-molecular-weight calcium-binding proteins of the S100 family (Wang et al., 2018). Human S100A8 and S100A9 contain 93 and 113 amino acids, respectively, and are predominantly expressed in myeloid-lineage cells, including neutrophils, monocytes, macrophages, and dendritic cells (Tardif et al., 2015). Under inflammatory or stress conditions, these proteins are released into the extracellular space and mainly function as the S100A8/A9 heterodimer or higher-order heterotetrameric complex, commonly referred to as calprotectin (Pruenster et al., 2016; Sun et al., 2024). Their EF-hand calcium-binding motifs and metal-binding capacity contribute to conformational stability, oligomerization, and interaction with intracellular or extracellular binding partners (Pruenster et al., 2016).

The biological relevance of S100A8/A9 in colorectal cancer (CRC) is closely linked to its role as an extracellular damage-associated molecular pattern (DAMP). Once released into the TME, S100A8/A9 can be recognized by pattern-recognition receptors, particularly TLR4 and the RAGE (Chen et al., 2024). Engagement of these receptors activates downstream signaling pathways, including MyD88-dependent nuclear factor-kappa B (NF-κB) and MAPK cascades (Schenten et al., 2018; Zhou et al., 2023), thereby providing a molecular basis for inflammation amplification, myeloid-cell recruitment, and tumor-promoting immune remodeling. Therefore, the structural organization of S100A8/A9 is not merely a biochemical feature but is directly associated with its capacity to act as a receptor-engaging inflammatory mediator in CRC.

Although S100A8 and S100A9 usually exert their biological functions as a heteromeric complex, the two subunits are not completely interchangeable. Available evidence suggests that S100A8 may be more closely associated with inflammatory signal amplification, cytoskeletal remodeling, and migration-related phenotypes, whereas S100A9 appears to have a stronger role in myeloid-cell regulation and immune suppression (Vogl et al., 2004; Markowitz and Carson, 2013; Shabani et al., 2018). However, these functional distinctions are context-dependent and should not be interpreted as strict separation of function, especially because most extracellular activities are mediated by the S100A8/A9 complex rather than by isolated monomers.

S100A8/A9 should also be considered within the broader S100 protein family. Human S100 proteins share conserved EF-hand calcium-binding motifs but differ in amino-acid sequence, hinge-region composition, oligomerization behavior, metal-binding properties, receptor preference, and target specificity (Bresnick et al., 2015; Gonzalez et al., 2020). At the sequence level, a comparative analysis of S100 family members reported that pairwise amino-acid sequence identity among different human S100 proteins ranges from approximately 16%–95%, with similarity values shown in brackets in the original analysis. In this analysis, S100A8 and S100A9 share approximately 27% sequence identity and 52% sequence similarity, indicating that their functional association cannot be explained by high sequence homology alone (Gross et al., 2014). Despite this limited sequence relatedness, several members—including S100A4, S100B, S100A6, S100P, and S100A12—engage RAGE/TLR4-associated signaling and have been implicated in inflammation, tumor invasion, immune regulation, or metastasis, indicating that functional convergence among S100 proteins arises more from shared receptor usage than from high sequence homology (Dahlmann et al., 2014; Yatime et al., 2016; Seguella et al., 2019; Carvalho et al., 2020).

This family-level perspective is important for interpreting the role of S100A8/A9 in CRC. Sequence homology does not necessarily indicate functional equivalence; however, shared receptor usage, especially through RAGE and TLR4, suggests that multiple S100 proteins may converge on overlapping inflammatory and tumor-promoting pathways. Therefore, S100A8/A9 should not be viewed as an isolated signaling molecule, but rather as a central component of a broader S100-family receptor network. This concept provides a structural and mechanistic rationale for later discussions on CRC progression, immune regulation, metastasis, and the potential compensatory effects of homologous S100 proteins during S100A8/A9-targeted therapy.

### 2.2 S100A8 and S100A9 as biomarkers of colorectal cancer

S100A8/A9 has been investigated as a biomarker in colorectal cancer, but its clinical significance should be interpreted in the context of tumor-associated inflammation rather than as a tumor-specific signal (Moris et al., 2016). In CRC, altered S100A8/A9 expression reflects both epithelial tumor biology and the activation state of the inflammatory and immune microenvironment. Its biomarker value can therefore be considered at three complementary levels: tissue expression patterns that capture local tumor-stroma and immune-cell interactions, biofluid-based detection that may support non-invasive screening or disease monitoring, and prognostic associations that link S100A8/A9 expression with tumor aggressiveness, metastasis, and immune subtype heterogeneity. This section summarizes these three lines of evidence while highlighting the potential and limitations of S100A8/A9 as a diagnostic, prognostic, and monitoring biomarker in CRC.

#### 2.2.1 Tissue-level expression patterns and clinical correlations

At the tissue level, S100A8/A9 expression in CRC is spatially heterogeneous rather than uniformly distributed across the tumor mass. Immunohistochemical studies have detected S100A8/A9-positive cells in both intratumoral and peritumoral regions, with marked enrichment at the invasive front and tumor-stroma interface (Başsorgun et al., 2014). These signals are mainly derived from infiltrating myeloid and inflammatory cells, including neutrophils, monocytes, and tumor-associated macrophages, although variable tumor-cell-associated staining has also been reported in some studies (Zha et al., 2016). This spatial pattern suggests that tissue S100A8/A9 is best interpreted as a marker of inflammatory tumor-stroma crosstalk rather than as a purely epithelial tumor-cell marker.

Clinicopathological studies further indicate that tissue S100A8/A9 expression is associated with aggressive CRC behavior. Duan et al. reported upregulation of S100A8 and S100A9 in more than half of CRC tissues, with higher expression correlating with histological differentiation, Dukes stage, and lymph node metastasis (Duan et al., 2013). Other protein-level analyses showed increased S100A9 expression in a substantial proportion of CRC samples compared with paired normal colonic mucosa, often accompanied by concordant S100A8 upregulation (Stulík et al., 1999). High S100A8/A9 expression has also been linked to larger tumor size, higher histological grade, metastatic status, and comparable expression patterns between primary tumors and matched metastatic lesions (Ang et al., 2010; Başsorgun et al., 2014). Collectively, these findings suggest that tissue S100A8/A9 marks an inflammation-enriched and metastasis-prone CRC phenotype.

Importantly, S100A8 and S100A9 may not carry identical clinical information. Ang et al. found that S100A8-positive stromal cells were reduced in Smad4-deficient CRC, whereas increased S100A9-positive cells were associated with larger tumor size (*p* = 0.0006), poorer differentiation (*p* = 0.036), and worse survival (*p* = 0.02) (Ang et al., 2010). This distinction suggests that S100A9 may provide stronger prognostic information than S100A8 in specific molecular or stromal contexts. However, these associations remain cohort-dependent and should be interpreted together with tumor stage, immune-cell composition, and molecular subtype.

#### 2.2.2 Biofluid-based detection and evaluation of diagnostic performance

At the biofluid level, serum S100A8 and S100A9 have generally been reported to be elevated in patients with CRC compared with healthy controls (Kim et al., 2009; Shu et al., 2016). In the study by Shu et al., both colon cancer and rectal cancer patients showed increased serum S100A8 and S100A9, with similar trends across the two tumor locations (Shu et al., 2016). These findings suggest that circulating S100A8/A9 is more likely to reflect a systemic inflammatory state associated with CRC than a tumor-site-specific signal.

The diagnostic value of serum S100A8/A9 is best understood as complementary to conventional tumor-burden markers rather than as a replacement for them. Carcinoembryonic antigen (CEA) remains the most widely used serum biomarker in CRC, but its sensitivity is limited, particularly in early-stage disease, and it does not reflect inflammatory or myeloid activation (Das et al., 2017). By contrast, circulating S100A8/A9 may add information on CRC-associated systemic inflammation. In receiver operating characteristic (ROC) analyses, combined serum S100A8 and S100A9 achieved area under the curve (AUC) values above 0.96 for colon and rectal cancer detection in the study by Shu et al. (Shu et al., 2016). In a 460-participant case-control study including 258 CRC patients, 99 patients with benign colonic disease, and 103 healthy donors, Zhou et al. reported that serum S100A9 outperformed CEA for CRC detection, with an AUC of 0.836 and a cut-off value of 18.22 ng/mL, compared with an AUC of 0.736 for CEA. A three-marker panel combining S100A9, tenascin-C (TNC), and CEA further increased the AUC to 0.908, with 79.8% sensitivity and 89.6% specificity, outperforming the conventional CEA plus carbohydrate antigen 19–9 (CA19-9) combination in that dataset (Zhou et al., 2019). Serum S100A9 also declined after surgery, suggesting possible value for postoperative monitoring. Overall, these findings support further evaluation of S100A9-based multi-marker models, while prospective validation is still needed before routine diagnostic or surveillance use.

Fecal detection should be considered separately from serum-based assays. In stool, S100A8/A9 is commonly measured as calprotectin, the stable heterodimeric complex of S100A8 and S100A9. Reported diagnostic cut-offs are approximately 50 μg/g or 10–20 mg/L, depending on the assay system (Johne et al., 2001; Kristinsson et al., 2001). However, fecal calprotectin is also elevated in inflammatory bowel disease, intestinal infection, and other inflammatory conditions. It is therefore better regarded as a surrogate marker of neutrophil-driven intestinal inflammation than as a tumor-specific CRC biomarker (Khaki-Khatibi et al., 2020).

#### 2.2.3 Functional relevance, prognostic significance, and subtype heterogeneity

Beyond their value as inflammatory biomarkers, S100A8 and S100A9 may also be functionally linked to CRC progression. *In vitro* studies showed that extracellular S100A8/A9 promotes CRC cell survival and migration (Duan et al., 2013), while experimental evidence further indicates that tumor-infiltrating monocytes and macrophages can induce S100A8/A9 expression in cancer cells and thereby enhance migration and invasion (Lim et al., 2016). In murine colon tumor models, S100A9 deficiency reduces tumor growth and metastatic burden, accompanied by decreased pro-inflammatory chemokine expression and reduced recruitment of CD11b^+^ Gr1^+^ myeloid cells in primary tumors and pre-metastatic organs (Ichikawa et al., 2011). These findings support S100A8/A9, particularly S100A9, as a functional component and biomarker of a pro-inflammatory, myeloid-enriched microenvironment. However, the functional evidence remains largely preclinical, whereas human biomarker data are mostly correlative.

The prognostic interpretation of S100A8/A9 is therefore context-dependent. High S100A9 expression, particularly in myeloid-rich tumor regions, is the more consistent correlate of advanced stage, immune suppression, and adverse outcome, whereas S100A8 shows weaker or compartment-dependent associations (Ang et al., 2010; Li et al., 2017). This suggests that the prognostic signal reflects a broader microenvironmental state, including chronic inflammation and myeloid accumulation, rather than tumor-cell-intrinsic expression alone. Accordingly, the clinical meaning of a S100A8/A9 signal depends on its cellular source, whether tumor cells, stromal cells, or infiltrating myeloid cells, and on the endpoint considered, including diagnosis, prognosis, metastasis, or treatment response. The two subunits should therefore not be treated as interchangeable.

This context-dependence is reinforced by molecular and immune-subtyping studies. Using multi-omics integration and single-cell transcriptomics, Bao et al. identified an immunosuppressive CRC subtype enriched in S100A9^+^ macrophages, in which S100A9^+^ macrophage accumulation was linked to impaired CD8^+^ T-cell effector function, poorer prognosis, and reduced immunotherapy responsiveness (Bao et al., 2023). S100A9 may thus be most informative as a biomarker in myeloid-enriched, immune-suppressed CRC rather than across all cases. In the preclinical component of the same study, PD-1 blockade combined with the S100A9 inhibitor tasquinimod improved anti-tumor responses, supporting the therapeutic relevance of this subtype-specific biomarker signal (Bao et al., 2023).

Taken together, S100A8/A9 is better viewed as a context-dependent functional biomarker axis than as a simple expression marker, integrating inflammatory activation, myeloid remodeling, metastatic propensity, and immune subtype. S100A9 shows the more consistent association with aggressive behavior and adverse prognosis, although this requires validation in larger, molecularly stratified cohorts. Its translational value therefore lies less in standalone diagnosis than in incorporation into biomarker panels for risk stratification, immune-subtype classification, and treatment-response monitoring.

## 3 Mechanisms of S100A8/A9 in the tumor microenvironment (TME) of CRC

Within the CRC tumor microenvironment, S100A8/A9 acts at multiple, interconnected levels—amplifying inflammation, establishing a myeloid-dominant immunosuppressive niche, and promoting invasion, angiogenesis, and metastasis. These major mechanisms, together with their principal signaling axes, downstream effectors, and the strength of supporting CRC evidence, are summarized in Table 1 and detailed in the following sections.

**TABLE 1:** Functional roles and signaling pathways of S100A8/A9 in colorectal cancer.

Functional role	Signaling axis	Principal downstream effectors	Consequence in CRC	References
Pro-inflammatory signaling	TLR4/RAGE → NF-κB and MAPK (p38, ERK1/2)	IL-6, TNF-α, IL-1β, CXCL1, CXCL5 and CCL5; miR-155 upregulation; inflammasome activation in colitis/CAC models	Amplifies chronic inflammatory signaling and supports inflammation-driven tumor-promoting immune remodeling	Ichikawa et al., 2011; Zha et al., 2016; Chen et al., 2024; Cho et al., 2024
Myeloid-cell recruitment and MDSC activation	S100A9–RAGE–p38 MAPK; S100A9–TLR4–NF-κB	MDSC chemotaxis and activation; Arg-1, iNOS, IL-10 and ROS upregulation	Promotes myeloid-dominant immune suppression and weakens CD8^+^ T-cell proliferation/function.	Cheng et al., 2008; Huang et al., 2019
S100A9^+^ macrophage enrichment and M2-like immunosuppression	TLR4/NF-κB/S100A9-associated macrophage polarization; S100A9+ macrophage-enriched immune subtype	M2-like TAM phenotype; S100A9^+^ macrophage enrichment; impaired CD8^+^ T-cell effector function	Defines a myeloid-enriched, immune-suppressed CRC subtype associated with poorer prognosis and reduced immunotherapy responsiveness	Hu et al., 2021; Bao et al., 2023
EMT and stromal–myeloid crosstalk	TGF-β/USF2/S100A8 axis; myofibroblast-derived IL-6/IL-8 induction of S100A8/A9 in infiltrating myeloid cells	EMT-related plasticity and migration/invasion programs; S100A8/A9 induction in tumor-infiltrating myeloid cells	Links inflammatory stromal–myeloid communication with tumor-cell migration, invasion and EMT-related phenotypes; direct S100A8/A9-driven CAF activation remains unproven	Kim et al., 2012; Lim et al., 2016; Li et al., 2021
Angiogenesis-related and pre-metastatic niche remodeling	RAGE/carboxylated-glycan signaling in colon tumor models; HIF-1/VEGF/S100A8-related vascular signaling in broader models	CXCL1, CCL5 and CCL7 induction; CD11b^+^Gr1^+^ myeloid-cell accumulation; VEGF-linked endothelial activation	Supports chemokine-mediated myeloid recruitment and metastatic niche formation; direct CRC-specific angiogenic evidence is weaker	Turovskaya et al., 2008; Ichikawa et al., 2011; Li et al., 2012; Ahn et al., 2014; Tsamchoe et al., 2023
Wnt/β-catenin modulation	S100A8/A9-associated β-catenin accumulation and nuclear translocation; upstream stabilization mechanism incompletely defined	c-MYC and MMP7 upregulation; β-catenin-dependent survival and migration	Acts as an inflammatory enhancer of β-catenin-dependent survival, migration and invasion rather than as a primary Wnt driver	Duan et al., 2013; Zhan et al., 2017; Zhao et al., 2022

Abbreviations: CRC, colorectal cancer; CAC, colitis-associated cancer; TAM, tumor-associated macrophage; MDSC, myeloid-derived suppressor cell; EMT, epithelial–mesenchymal transition; PMN, pre-metastatic niche; TLR4, Toll-like receptor 4; RAGE, receptor for advanced glycation end products; MAPK, mitogen-activated protein kinase; HIF-1, hypoxia-inducible factor 1; VEGF, vascular endothelial growth factor.

### 3.1 Effects of S100A8/A9 on the pro-inflammatory response within the TME

Although S100A8/A9 is a prototypical damage-associated molecular pattern (DAMP), its inflammatory activity is context-dependent rather than strictly unidirectional. During acute or resolving inflammation, redox-sensitive modifications of S100A8/A9, including oxidation and S-nitrosylation, can limit excessive leukocyte activation and tissue injury (Lim et al., 2011; Wang et al., 2018). In the persistently inflamed colorectal cancer tumor microenvironment (TME), however, these restraining effects are outweighed, and S100A8/A9 mainly acts as an amplifier of chronic pro-inflammatory signaling.

Mechanistically, extracellular S100A8/A9 engages TLR4 and the RAGE, activating NF-κB and MAPK pathways, including extracellular signal-regulated kinase 1/2 (ERK1/2) and p38 MAPK. These pathways promote the production of inflammatory cytokines and chemokines, including interleukin-6 (IL-6), tumor necrosis factor-alpha (TNF-α), interleukin-1β(IL-1β), CXCL1, CXCL5, and CCL5, thereby sustaining myeloid-cell recruitment and tumor-promoting inflammation (Ichikawa et al., 2011; Chen et al., 2024). In macrophages, S100A8 activates NF-κB signaling and upregulates miR-155 expression, thereby increasing IL-1β and TNF-α secretion and enhancing CRC-cell migration (Zha et al., 2016). These findings support an inflammatory-amplification axis, but do not establish a miR-155-dependent feedback loop in CRC.

Additional CRC-specific evidence links S100A8/A9 to inflammation-driven tumorigenesis. Zhang et al. showed that inflammation-induced S100A8 promotes colorectal tumorigenesis through the Akt1-Smad5-Id3 axis (Zhang et al., 2015). Hu et al. further connected microbial inflammation to this axis by showing that *Fusobacterium* nucleatum induces S100A9 expression through TLR4/NF-κB signaling in the CRC microenvironment (Hu et al., 2021). In colitis-associated CRC models, colon-targeted blockade of S100A8/A9 interactions with TLR4 and RAGE reduced inflammatory signaling, attenuated colitis, and decreased tumor burden, further supporting the functional relevance of this receptor-mediated inflammatory axis (Cho et al., 2024).

Taken together, S100A8/A9 sustains chronic inflammation in the CRC TME by linking DAMP signaling, TLR4/RAGE activation, NF-κB/MAPK-related cytokine induction, and chemokine-mediated myeloid-cell recruitment. This inflammatory axis contributes to the transition from chronic intestinal inflammation to tumor-promoting immune remodeling and provides a mechanistic rationale for receptor-level or pathway-level therapeutic intervention.

### 3.2 Immune regulation: S100A8/A9 shapes an immune-tolerant TME

In CRC, the immunological significance of S100A8/A9 is most evident in its regulation of myeloid-cell recruitment and function. Rather than acting as a general immune suppressor in all settings, S100A8/A9 marks and reinforces a myeloid-enriched tumor microenvironment characterized by MDSC accumulation, S100A9^+^macrophage enrichment, and myeloid-mediated impairment of T-cell activity (Huang et al., 2019; Bao et al., 2023). Its immunological role should therefore be interpreted according to cellular source, dominant myeloid population, and CRC molecular subtype.

The best-supported mechanism involves S100A9-mediated regulation of MDSCs. Huang et al. reported that both S100A9 and MDSCs were elevated in CRC tumor tissues and peripheral blood and were associated with disease progression. Mechanistically, S100A9 promoted MDSC chemotaxis through the RAGE-p38 MAPK pathway and enhanced MDSC activation via TLR4-NF-κB signaling. This activation increased immunosuppressive mediators, including arginase-1 (Arg-1), inducible nitric oxide synthase (iNOS), interleukin-10 (IL-10), and reactive oxygen species (ROS), thereby strengthening MDSC-mediated suppression of CD8^+^T-cell proliferation. TLR4 blockade attenuated this effect (Cheng et al., 2008; Huang et al., 2019). These findings provide direct CRC-specific evidence that S100A9 promotes immune tolerance mainly through myeloid-cell recruitment and activation.

S100A8/A9 expression in CRC cell models may further support this myeloid-rich milieu by inducing inflammatory cytokine and chemokine programs. Overexpression of S100A8/A9 activates signal transducer and activator of transcription 3 (STAT3), NF-κB, and ERK/MAPK signaling and increases the secretion of mediators such as granulocyte-macrophage colony-stimulating factor (GM-CSF), IL-1α, IL-1β, CCL3, and CXCL5 (Chen et al., 2024). These signals may favor inflammatory-cell recruitment and tumor-promoting immune remodeling, and they also help shape the macrophage compartment described below. However, evidence for a defined S100A8/A9-driven autocrine-paracrine feedback loop between tumor cells and MDSCs remains incomplete.

S100A9 also shapes the macrophage compartment, where it can drive polarization of tumor-associated macrophages toward an immunosuppressive M2-like phenotype that favors a more malignant tumor-cell phenotype (Hu et al., 2021). Consistently, multi-omics and single-cell analyses have identified an immunosuppressive CRC subtype enriched in S100A9^+^ macrophages. This subtype is associated with impaired CD8^+^ T-cell effector function, poorer prognosis, and reduced immunotherapy responsiveness (Bao et al., 2023). These findings provide a rationale for combining S100A9 inhibition with immune checkpoint blockade, as discussed in Section 4.

Extracellular vesicle (EV)-associated S100A9 may represent an additional systemic indicator of myeloid immune remodeling. In patients with colorectal cancer liver metastasis, circulating EVs containing S100A9 were associated with histopathological growth pattern, immune phenotype, and therapeutic response (Tsamchoe et al., 2023). However, direct evidence that S100A8/A9-containing EVs actively drive immune suppression in CRC remains limited and requires mechanistic validation.

Taken together, S100A8/A9 shapes the CRC immune microenvironment primarily through myeloid-centered mechanisms. The strongest evidence supports S100A9-mediated MDSC recruitment and activation via RAGE-p38 MAPK and TLR4-NF-κB signaling, together with S100A9^+^macrophage enrichment in immune-suppressed subtypes. Proposed roles in direct T-cell exhaustion, dendritic-cell dysfunction, and EV-driven systemic remodeling remain less directly supported and should be interpreted cautiously. S100A8/A9 is therefore best viewed as a context-dependent regulator and biomarker of myeloid-dominant immune suppression rather than a universal immune switch in CRC.

### 3.3 Relationship between S100A8/A9 and the tumor metastatic phenotype

**FIGURE 1:**
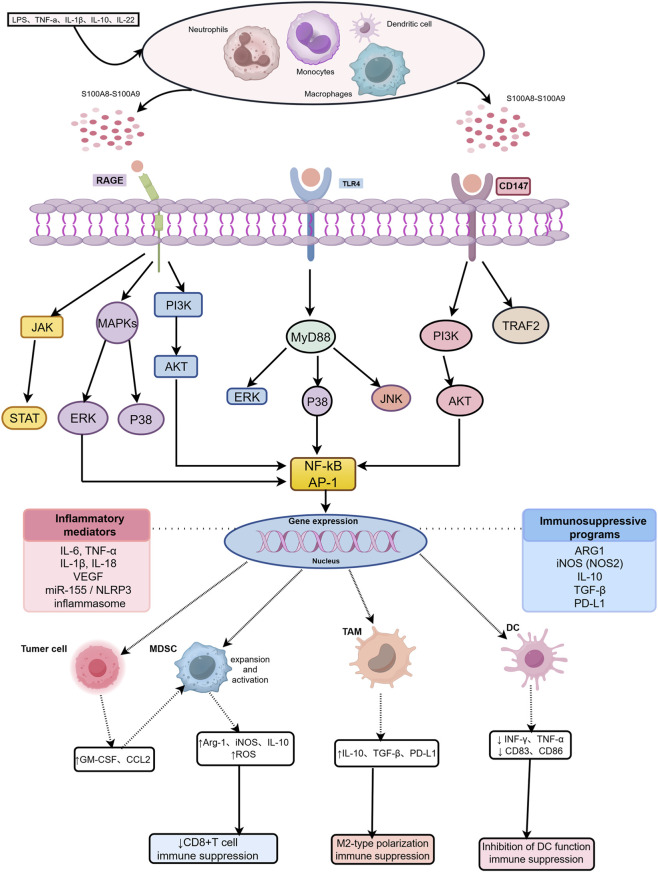
Mechanisms of S100A8/A9 in the inflammatory and immune microenvironment of CRC (Created by Figdraw).

**FIGURE 2:**
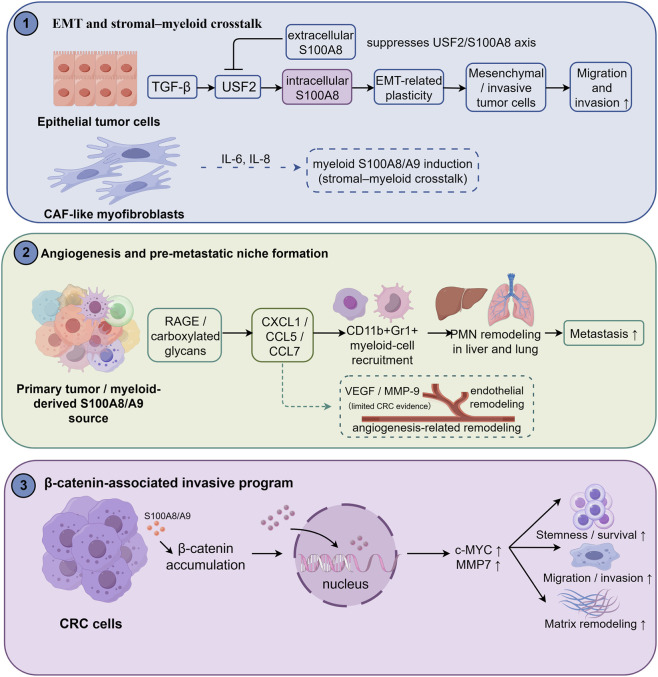
S100A8/A9-driven invasion and metastasis in CRC (Created by Figdraw).

Beyond its inflammatory and immunoregulatory roles, S100A8/A9 is increasingly implicated in CRC invasion and metastatic remodeling. As outlined in Figure 1, S100A8/A9 connects inflammatory signaling and myeloid-dominant immune suppression within the CRC tumor microenvironment. Building on this inflammatory-immune framework, Figure 2 focuses on the invasion-metastasis module, in which S100A8/A9 couples myeloid inflammation with tumor-cell plasticity, stromal communication, chemokine-driven niche formation, and Wnt/β-catenin-dependent invasive programs. The strength of evidence, however, is uneven across these mechanisms. Direct CRC evidence is strongest for S100A8/A9-associated tumor-cell migration, S100A9-dependent myeloid recruitment, and β-catenin-mediated survival and motility, whereas direct roles in CAF activation and angiogenesis remain less firmly established.

This section therefore discusses three interconnected but mechanistically distinct layers of S100A8/A9-associated metastasis: EMT and stromal-myeloid crosstalk, angiogenesis-related and pre-metastatic niche remodeling, and modulation of Wnt/β-catenin signaling. This organization helps distinguish experimentally supported CRC mechanisms from plausible but less validated extrapolations.

#### 3.3.1 EMT and stromal‐myeloid crosstalk

S100A8/A9 is linked to CRC invasion and migration through both tumor-cell-intrinsic and microenvironment-mediated mechanisms. Tumor-infiltrating monocytes and macrophages can induce S100A8/A9 expression in cancer cells, which in turn enhances tumor-cell migration and invasion (Lim et al., 2016). This positions S100A8/A9 not simply as an epithelial marker, but as a mediator of inflammatory tumor-stroma crosstalk.

More direct evidence for epithelial-mesenchymal transition (EMT) regulation comes from the TGF-β/USF2/S100A8 axis. Li et al. showed that transforming growth factor-β(TGF-β) upregulates upstream transcription factor 2 (USF2), which transcriptionally activates S100A8 in CRC cells. Intracellular S100A8 then promotes migration, invasion, EMT, and metastasis *in vitro* and *in vivo*, whereas extracellular S100A8 suppresses the same USF2/S100A8 axis (Li et al., 2021). This compartment-dependent behavior indicates that intracellular and extracellular S100A8 should not be interpreted as functionally equivalent in EMT.

The relationship between S100A8/A9 and cancer-associated fibroblasts (CAFs) appears to be indirect rather than a direct S100A8/A9-driven CAF-activation loop. Available data support a stromal-myeloid model in which CAF-like myofibroblasts secrete interleukin-6 (IL-6) and interleukin-8 (IL-8), inducing S100A8/A9 expression in tumor-infiltrating myeloid cells and favoring their differentiation toward MDSC-like or M2-like phenotypes (Kim et al., 2012). Direct evidence that S100A8/A9 itself activates CAFs through TGF-β/STAT3 signaling in CRC remains lacking.

Taken together, S100A8/A9 contributes to CRC invasion mainly by coupling inflammatory myeloid infiltration to tumor-cell migration and EMT-related programs. The strongest evidence supports an intracellular S100A8-TGF-β/USF2 axis driving EMT and stromal-myeloid IL-6/IL-8 signaling that induces S100A8/A9 in infiltrating myeloid cells. By contrast, a direct S100A8/A9-CAF activation loop should be regarded as unproven in CRC.

#### 3.3.2 Promotion of angiogenesis and pre-metastatic niche formation

S100A8/A9 may contribute to CRC metastatic progression by linking inflammatory myeloid remodeling with angiogenesis and pre-metastatic niche (PMN) formation (Ichikawa et al., 2011; Peinado et al., 2017). However, the strength of evidence differs across these processes. Direct CRC-relevant data are strongest for chemokine-mediated myeloid recruitment and PMN remodeling, whereas evidence for angiogenesis is derived mainly from broader vascular and myeloid-cell models.

The most direct CRC-relevant evidence comes from colon tumor models. Ichikawa et al. showed that myeloid-derived S100A8/A9 interacts with the RAGE and carboxylated glycans on colon tumor cells, activating pro-tumorigenic gene programs that include inflammatory chemokines such as CXCL1, CCL5, and CCL7 (Turovskaya et al., 2008; Ichikawa et al., 2011). In S100A9-deficient mice, tumor growth and metastasis were reduced, serum CXCL1 elevation was blunted, and CD11b^+^Gr1^+^myeloid-cell accumulation decreased in both primary tumors and pre-metastatic liver and lung niches (Ichikawa et al., 2011). These findings support a model in which S100A8/A9 promotes metastatic competence primarily through chemokine induction and myeloid-cell recruitment, rather than through a fully established vascular endothelial growth factor (VEGF)/matrix metalloproteinase-9 (MMP-9) angiogenic cascade in CRC.

Evidence for a direct angiogenic role is weaker and largely non-CRC. Low concentrations of S100A8/A9 can promote vascular endothelial cell proliferation, migration, and tube formation *in vitro* (Li et al., 2012; Zhong et al., 2020). In addition, myeloid hypoxia-inducible factor-1 (HIF-1) signaling has been linked to VEGF- and S100A8-dependent neovascularization in broader vascular models (Ahn et al., 2014). These findings make a pro-angiogenic role biologically plausible, but they should be interpreted as general vascular evidence rather than CRC-specific proof.

S100A8/A9 has also been implicated in PMN formation in broader cancer models. Primary-tumor-derived soluble factors can induce S100A8/A9 expression in pre-metastatic lung endothelial and myeloid cells, attracting Mac-1^+^ myeloid cells and creating a permissive metastatic microenvironment (Hiratsuka et al., 2006; Peinado et al., 2017). Consistent with a systemic correlate in CRC, circulating extracellular vesicles (EVs) containing S100A9 have been detected in patients with colorectal liver metastasis and were associated with histopathological growth pattern, immune phenotype, and therapeutic response (Tsamchoe et al., 2023). However, EV-associated S100A9 should currently be viewed mainly as a metastatic immune marker rather than as proven evidence that S100A8/A9-containing EVs drive PMN formation in CRC.

Taken together, S100A8/A9 appears to promote CRC metastasis mainly by coordinating chemokine programs, myeloid-cell recruitment, and PMN remodeling. Its role in angiogenesis is biologically plausible, but direct CRC-specific evidence linking S100A8/A9 to microvessel density, VEGF expression, or MMP-9-driven angiogenesis remains limited. This distinction should be preserved to avoid overstating the mechanistic certainty of S100A8/A9-driven angiogenesis in CRC.

#### 3.3.3 Regulation of tumor invasion and metastatic phenotype through the Wnt/β-catenin pathway

Wnt/β-catenin signaling is a central driver of CRC initiation, stem-cell maintenance, invasion, and therapeutic resistance (Zhao et al., 2022). Although alterations in adenomatous polyposis coli (APC) or the β-catenin gene CTNNB1 are the dominant genetic basis of Wnt activation in CRC, pathway output is not fixed by mutation alone; it can be further shaped by ligand availability and microenvironmental cues, including stromal Wnt signals (Voloshanenko et al., 2013; Aizawa et al., 2019). In this context, S100A8/A9 appears to act as an inflammatory modifier of Wnt activity rather than as a primary genetic driver of the pathway.

The most direct CRC evidence comes from Duan et al. In HCT116 and SW480 cells, recombinant S100A8 or S100A9 increased total and nuclear β-catenin and upregulated the canonical β-catenin target genes c-MYC and matrix metalloproteinase-7 (MMP7); functionally, S100A8/A9 enhanced CRC-cell viability and migration, whereas β-catenin knockdown partially reversed these effects (Duan et al., 2013). These data support a β-catenin-dependent contribution of extracellular S100A8/A9 to CRC-cell survival and motility. However, this study did not fully define how S100A8/A9 stabilizes β-catenin, and direct evidence for regulation of the glycogen synthase kinase 3β (GSK3β) destruction complex remains incomplete (Stamos and Weis, 2013; Katoh and Katoh, 2022).

The biological relevance of this axis lies in its ability to connect inflammatory signaling with invasive and stem-like phenotypes. c-MYC supports proliferative and stemness-related programs, whereas MMP7 contributes to extracellular-matrix remodeling and invasion (Duan et al., 2013; Zhan et al., 2017). Thus, S100A8/A9-induced β-catenin activation may provide one route through which a myeloid-rich inflammatory microenvironment reinforces tumor-cell plasticity and metastatic competence. This mechanism should not, however, be overstated as the dominant route of Wnt regulation in CRC, where canonical pathway mutations and stromal Wnt ligands also make major contributions.

The proposed interaction between S100A8/A9 and cancer-associated fibroblast (CAF)-derived Wnt signals should therefore be framed cautiously. CAF-derived Wnt2 promotes CRC progression and represents a plausible microenvironmental amplifier of β-catenin signaling (Aizawa et al., 2019); however, direct evidence that S100A8/A9 establishes a CAF-Wnt2-S100A8/A9 positive feedback loop in CRC is currently lacking. Likewise, links between β-catenin activation and immunosuppressive programs mediated by IL-10, CCL2, MDSCs, or regulatory T cells remain biologically plausible but indirect in the S100A8/A9–CRC setting.

Taken together, S100A8/A9 is best viewed as an inflammatory enhancer of Wnt/β-catenin-dependent survival, migration, and invasion rather than as an independent master regulator of the Wnt pathway. This interpretation integrates inflammation, stromal cues, and metastatic behavior while preserving the distinction between demonstrated CRC evidence and mechanistic extrapolation.

### 3.4 Functional overlap with other S100 family members in CRC progression

S100A8/A9 should be interpreted within the broader S100-family signaling network rather than as an isolated mediator. Depending on protein structure and cellular context, extracellular S100 proteins can engage several cell-surface receptors, including RAGE, TLR4, EGFR, scavenger receptors, and G protein-coupled receptors (Donato et al., 2013; Gonzalez et al., 2020). This receptor-level convergence is relevant to CRC because several S100 family members activate overlapping downstream cascades, suggesting that shared receptor usage, rather than sequence homology alone, may explain why different S100 members contribute to similar tumor-promoting processes.

Several examples illustrate this convergence in CRC. Extracellular S100A4 binds RAGE and promotes CRC-cell migration and invasion through MAPK/ERK- and hypoxia-associated signaling, and high tumor RAGE expression has been associated with metachronous metastasis and poorer survival (Dahlmann et al., 2014). S100P similarly engages RAGE to stimulate colon cancer-cell growth and migration through ERK1/2 and NF-κB signaling (Fuentes et al., 2007). Enteric glia-derived S100B can likewise activate RAGE-dependent signaling in colon adenocarcinoma cell models, promoting proliferation, invasiveness, and vascular endothelial growth factor (VEGF)-associated angiogenic responses (Seguella et al., 2019). Beyond this RAGE-centered axis, the family also shows pathway-level diversity: S100A6, for example, is itself a transcriptional target of β-catenin and is upregulated in CRC cell models (Kilańczyk et al., 2012).

These observations indicate that CRC progression may engage a broader S100-family receptor network in which different members partially converge on inflammation, invasion, angiogenesis, and metastatic remodeling. This carries therapeutic relevance, because selective inhibition of S100A8/A9 may not fully suppress S100-family-driven signaling if other RAGE- or TLR4-engaging ligands remain active in the tumor microenvironment (see Section 4.4). However, direct evidence that other S100 proteins can compensate for S100A8/A9 inhibition in CRC remains limited; functional redundancy should therefore be presented as a mechanistic hypothesis and a rationale for receptor-level or combination strategies, rather than as an established CRC resistance mechanism.

## 4 S100A8/A9-related therapeutic strategies

**FIGURE 3:**
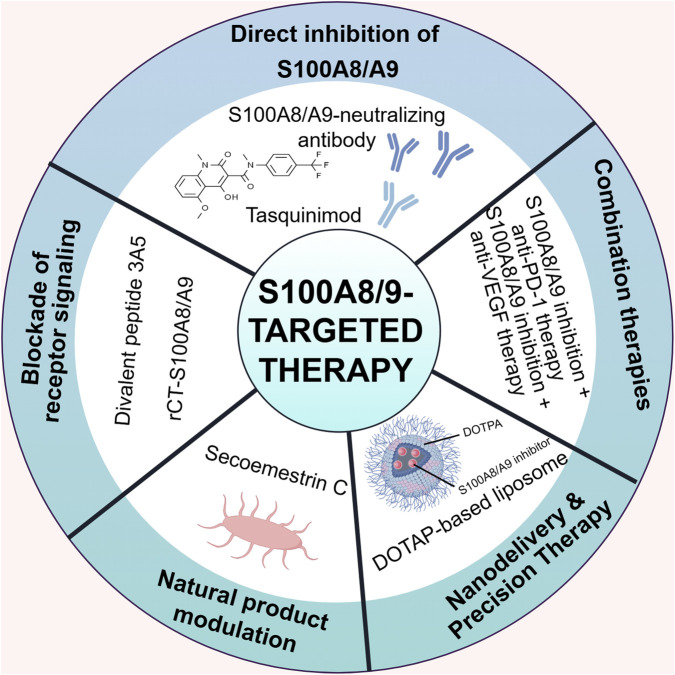
S100A8/A9-targeted therapeutic strategies in CRC (Created by Figdraw).

Given its role in sustaining inflammation, shaping an immunosuppressive microenvironment, and promoting metastatic progression in CRC, the S100A8/A9 axis has attracted increasing interest as a therapeutic target. Most current evidence remains preclinical, but available studies suggest that this pathway may be modulated at several levels: direct inhibition or neutralization of extracellular S100A8/A9, blockade of receptor-mediated signaling through RAGE/TLR4 and downstream NF-κB, MAPK, or STAT3 pathways, remodeling of the tumor immune microenvironment through combination therapy, and emerging precision-delivery or biologic approaches. These strategies are summarized in Figure 3 and Table 2 and discussed below.

**TABLE 2:** Therapeutic strategies targeting the S100A8/A9 axis in colorectal cancer and related tumor models.

Strategy/agent	Category	Mechanism/target	Model and key findings	Translational status	References
Anti-S100A9 antibody	Direct neutralization	Neutralizes extracellular S100A9 and lowers pro-inflammatory cytokine production	DSS colitis and AOM/DSS colitis-associated cancer models; reduced tumor growth/incidence, immune-cell infiltration and TNF-α/IL-1β/IL-6 production	Preclinical evidence in CAC; not validated in sporadic, metastatic or treatment-resistant CRC.	Zhang et al. (2017)
Tasquinimod	Small-molecule S100A9-associated inhibitor	Quinoline-3-carboxamide agent associated with S100A9 targeting; interferes with S100A9-linked RAGE/TLR4 inflammatory and myeloid-regulatory signaling	Preclinical tumor models show myeloid remodeling, reduced angiogenesis and enhanced anti-tumor immunity; mCRPC phase III improved rPFS without OS benefit; phase II studies in other solid tumors and a relapsed/refractory myeloma meeting report provide non-CRC clinical experience	Clinical experience is largely non-CRC; pharmacologically feasible but not CRC-proven	Mehta and Armstrong, 2016; Gupta et al., 2014; Olsson et al., 2015; Shen et al., 2015; Sternberg et al., 2016; Escudier et al., 2017; Vogl et al., 2024
Divalent peptide 3A5	Receptor-blocking peptide (TLR4/MD-2)	Competitively binds the TLR4/MD-2 complex and suppresses S100A8-induced IL-8 and VEGF production	SW480 xenograft models showed dose-dependent tumor suppression; syngeneic models showed improved anti-PD-1 efficacy, suppressed pulmonary tumor-cell recruitment/metastatic niche formation and increased CD8^+^ T-cell infiltration	Preclinical CRC/colon-tumor evidence	Deguchi et al. (2023)
rCT-S100A8/A9	Dual-receptor blocking peptide (TLR4 and RAGE)	Colon-targeted peptide system that inhibits S100A8/A9 engagement of both TLR4 and RAGE.	DSS colitis, AOM/DSS CAC and oxaliplatin-resistant CRC xenograft models; suppressed inflammasome activation and reduced tumor burden	Preclinical CRC/CAC evidence; possible relevance to chemoresistant disease	Cho et al. (2024)
Secoemestrin C (Sec C)	Natural product	Downregulates S100A8 and acts on the p38-S100A8 feed-forward regulatory loop	CRC stem-cell models and HCT8 xenografts; reduced proliferation, self-renewal, migration and oxaliplatin-resistant growth	Early preclinical concept; single-study evidence and no clinical CRC validation	Zhou et al. (2024)
Tasquinimod + anti-PD-1	Combination therapy	Targets S100A9-associated myeloid suppression together with immune-checkpoint blockade	S100A9^+^ macrophage-enriched CRC models; improved anti-tumor responses by remodeling the immunosuppressive myeloid compartment and enhancing T-cell-mediated immunity	Preclinical CRC evidence; supports biomarker-guided combination rather than unselected monotherapy	Bao et al. (2023)
3A5 + bevacizumab or anti-PD-1	Combination therapy	Combines S100A8-TLR4/MD-2 blockade with anti-angiogenic or immune-checkpoint treatment	3A5 synergized with bevacizumab in SW480 xenografts and enhanced anti-PD-1 efficacy in syngeneic models	Preclinical CRC/colon-tumor evidence	Deguchi et al. (2023)
Anti-S100A8 scFv antibody	Biologic engineering	Single-chain variable fragment antibody binds S100A8 and suppresses S100A8-induced inflammatory signaling	*In vitro* activity in macrophages and HT-29 CRC cells	*In vitro* proof of concept; requires CRC-relevant animal validation	Raeisi et al. (2025)
DOTAP-based nanoliposomes	Nanodelivery/myeloid modulation platform	Cationic-lipid carrier associated with reduced S100A8 and Arg-1 expression in splenic MDSCs	C26 colon cancer model; reduced splenic MDSC population/activity and S100A8/Arg-1 expression	Preclinical delivery proof of concept; not yet a tumor-targeted S100A8/A9 inhibitor platform	Taheri et al. (2022)
Broad systemic S100A8/A9 or S100A9 blockade	Safety/context-dependence caveat	Systemic perturbation of S100A8/A9 or blockade of extracellular S100A9-TLR4 signaling may disrupt beneficial early anti-tumor immune recruitment	CT26 and broader mouse-model evidence suggests that indiscriminate blockade can increase tumor growth, weaken anti-PD-L1 efficacy or promote protumorigenic myelopoiesis	Cautionary preclinical evidence; supports tissue-selective delivery and biomarker-guided patient selection	Demir et al., 2024; Luo et al., 2025

Abbreviations: CRC, colorectal cancer; CAC, colitis-associated cancer; DSS, dextran sodium sulfate; AOM, azoxymethane; MDSC, myeloid-derived suppressor cell; TLR4, Toll-like receptor 4; RAGE, receptor for advanced glycation end products; MD-2, myeloid differentiation factor 2; VEGF, vascular endothelial growth factor; PD-1, programmed cell death protein 1; PD-L1, programmed death-ligand 1; mCRPC, metastatic castration-resistant prostate cancer; rPFS, radiographic progression-free survival; OS, overall survival; IRd, ixazomib, lenalidomide and dexamethasone; scFv, single-chain variable fragment; Arg-1, arginase-1.

### 4.1 Direct targeting of S100A8/A9

Direct targeting of S100A8/A9 aims to neutralize extracellular S100A8/A9 or to prevent S100A9-mediated activation of inflammatory and myeloid-immunosuppressive signaling. Among antibody-based approaches, anti-S100A9 antibodies have provided proof-of-concept evidence in inflammation-associated colorectal tumorigenesis. In dextran sodium sulfate (DSS)-induced colitis and azoxymethane (AOM)/DSS-induced colitis-associated cancer (CAC) models, anti-S100A9 antibody treatment delayed tumor growth and reduced tumor incidence, accompanied by decreased immune-cell infiltration and lower production of pro-inflammatory cytokines such as TNF-α, IL-1β, and IL-6 (Zhang et al., 2017; Xia et al., 2024). However, this evidence remains confined to animal models of colitis-associated cancer, and its relevance to sporadic, metastatic, or treatment-resistant CRC has not yet been established.

Tasquinimod is the most extensively studied small-molecule agent associated with S100A9 targeting (Mehta and Armstrong, 2016). This quinoline-3-carboxamide derivative binds the metal-ion-binding site of S100A9 and interferes with its engagement of RAGE and TLR4, thereby dampening downstream inflammatory and myeloid-regulatory signaling (Gupta et al., 2014). In preclinical tumor models it remodels the myeloid compartment, reduces angiogenesis, and enhances anti-tumor immune activity, acting as an immune-modulating rather than a conventional cytotoxic agent (Olsson et al., 2015; Shen et al., 2015; Shen and Pili, 2019).

Clinical experience with tasquinimod remains largely outside CRC. In metastatic castration-resistant prostate cancer, phase III evaluation improved radiographic progression-free survival but did not yield an overall-survival benefit (Sternberg et al., 2016), and trials in other solid tumors provided safety and pharmacodynamic data without establishing monotherapy efficacy (Osanto et al., 2013; Escudier et al., 2017). More recently, on the rationale that myeloid-derived suppressor cell (MDSC)-derived S100A9 sustains an immunosuppressive microenvironment, a phase 1b trial evaluated tasquinimod alone and in combination with ixazomib, lenalidomide, and dexamethasone (IRd) in relapsed/refractory multiple myeloma, with preliminary reports indicating acceptable tolerability and possible anti-myeloma activity in heavily pretreated patients (Vogl et al., 2024). This experience supports pharmacological feasibility but should be read as translational support rather than proof of CRC efficacy.

The combination rationale is particularly relevant to CRC. Bao et al. identified an immunosuppressive CRC subtype enriched in S100A9^+^macrophages and showed that combining PD-1 blockade with tasquinimod enhanced anti-tumor responses in preclinical CRC models (Bao et al., 2023). This suggests that tasquinimod may be more useful as a microenvironment-remodeling agent in selected immune-suppressed CRC subtypes than as monotherapy in unselected patients, and that future CRC development will depend on molecular stratification and rational combinations rather than single-agent use.

Despite this rationale, direct targeting of S100A8/A9 faces several constraints. Most CRC evidence remains preclinical, and tasquinimod has not yet been clinically validated in CRC patients. Because S100A8/A9 also participates in host defense and inflammation resolution, broad systemic inhibition may disrupt normal myeloid and immune function (Demir et al., 2024; Luo et al., 2025). Functional redundancy within the S100 family may further limit single-ligand targeting, as other S100 proteins can converge on shared receptors such as RAGE or TLR4 (Bresnick et al., 2015; Gonzalez et al., 2020). These limitations argue for biomarker-guided patient selection, tumor- or cell-selective delivery, and rational combinations rather than non-selective systemic monotherapy.

### 4.2 Blocking receptor signaling pathways

Beyond directly targeting S100A8/A9, blocking its receptor engagement and downstream signaling represents a second strategy (Deguchi and Maru, 2025). Exploiting the S100A8-TLR4/MD-2 binding interface, Deguchi et al. designed a divalent peptide, 3A5, that competitively binds the TLR4/MD-2 complex and suppresses S100A8-induced cytokine and chemokine production, including IL-8 and VEGF, in colorectal tumor cells (Deguchi et al., 2023). In SW480 xenograft models, 3A5 inhibited tumor growth in a dose-dependent manner and acted synergistically with the anti-VEGF antibody bevacizumab. In syngeneic tumor models, 3A5 further enhanced the efficacy of anti-PD-1 therapy, accompanied by increased intratumoral CD8^+^ T-cell infiltration. It also suppressed pulmonary tumor-cell recruitment and pre-metastatic niche formation (Deguchi et al., 2023).

Extending this to dual-receptor blockade, Cho et al. developed a colon-targeted peptide system (rCT-S100A8/A9) that simultaneously inhibits S100A8/A9 engagement of both TLR4 and RAGE (Cho et al., 2024). Delivered via intestinal targeting, it suppressed inflammasome activation and reduced tumor burden in DSS-induced colitis and AOM/DSS-induced CAC models, and notably retained anti-tumor activity in oxaliplatin-resistant CRC xenografts, pointing to a possible role in chemoresistant disease (Cho et al., 2024).

Together, these studies provide preclinical proof of concept that receptor-level blockade can reproduce the anti-tumor effects of S100A8/A9 inhibition and extend them through rational combinations. As with direct inhibitors, however, the evidence remains experimental, and because RAGE and TLR4 also mediate physiological immune signaling, the selectivity and long-term safety of sustained receptor blockade remain to be established.

### 4.3 Natural products regulating S100A8/A9 signaling

Natural products may offer an additional route for modulating S100A8/A9-related inflammatory signaling, although direct CRC evidence remains limited (Rejhová et al., 2018; Wang et al., 2022). The best-characterized example is secoemestrin C (Sec C), a fungal-derived natural compound that has been reported to suppress colorectal cancer stem cell (CSC)-like phenotypes through the p38-S100A8 feed-forward regulatory loop (Zhou et al., 2024). In CRC models, Sec C reduced CSC proliferation, self-renewal, migration, and oxaliplatin-resistant cell growth by downregulating S100A8; anti-tumor activity was also observed in HCT8 xenografts without obvious toxic effects in major organs at the tested doses (Zhou et al., 2024). These findings position S100A8 as a link between inflammatory signaling, CSC maintenance, and chemoresistance, and support Sec C as an early proof of concept for pharmacologic S100A8 modulation. However, the evidence rests on a single preclinical study, and Sec C has not been clinically validated in CRC. Natural-product modulation of S100A8/A9 should therefore be framed as an early-stage concept rather than an established therapeutic strategy.

### 4.4 Combination therapy and future perspectives

Because S100A8/A9 participates in inflammation, myeloid-cell recruitment, immune suppression, angiogenesis, and metastatic remodeling, single-agent inhibition is unlikely to fully reverse the pro-tumorigenic CRC microenvironment. This limitation may be amplified by functional overlap within the S100 family, as discussed in Section 3.4. Several extracellular S100 proteins can engage overlapping inflammatory receptor networks, particularly RAGE and, in some contexts, TLR4, suggesting that S100A8/A9 blockade alone may be bypassed by related S100 ligands or convergent receptor-level signaling (Björk et al., 2013; Bresnick, 2018). However, direct evidence that other S100 proteins compensate for S100A8/A9 inhibition in CRC remains limited. This issue should therefore be framed as a rationale for combination or receptor-level strategies rather than as an established resistance mechanism.

Current CRC-relevant evidence supports combination therapy mainly in preclinical settings. In S100A9^+^macrophage-enriched CRC models, tasquinimod combined with PD-1 blockade improved anti-tumor responses by remodeling the immunosuppressive myeloid compartment and enhancing T-cell-mediated immunity (Bao et al., 2023). Similarly, S100A8-targeting peptide strategies have been reported to improve the activity of anti-angiogenic or immune-checkpoint approaches in experimental CRC models, supporting the idea that S100A8/A9 blockade may be most effective when paired with therapies that target angiogenesis or immune escape (Deguchi et al., 2023). These findings suggest that the most rational use of S100A8/A9-targeted therapy in CRC may not be monotherapy, but biomarker-guided combination treatment in myeloid-enriched or immune-suppressed tumors.

Evidence from non-CRC malignancies further supports the broader therapeutic logic, but should not be interpreted as CRC validation. In multiple myeloma, tasquinimod has been shown to target immunosuppressive myeloid cells and reduce tumor-supportive signaling (Fan et al., 2023). In hepatocellular carcinoma, S100A9^+^CD14^+^monocytes have been linked to resistance to anti-PD-1 immunotherapy by attenuating T-cell-mediated antitumor function (Tu et al., 2024). These findings provide translational precedents for targeting S100A9-associated myeloid suppression, but CRC-specific clinical efficacy remains unproven. Future work should prioritize molecular patient stratification, rational pairing with immune checkpoint blockade or anti-angiogenic therapy, and tumor- or tissue-selective delivery systems. Intervention strategies targeting S100A8/A9 may provide a rationale for future precision-oriented therapeutic development in CRC.

### 4.5 Nanomedicine-based delivery and prospects for precision therapy

Although S100A8/A9 is widely regarded as a pro-inflammatory and immunosuppressive mediator in CRC, therapeutic blockade of this axis should be interpreted in a context-dependent manner. In the CT26 tumor model, paquinimod-mediated blockade of extracellular S100A9-TLR4 signaling increased tumor growth, reduced tumor immune infiltration, and weakened the efficacy of anti-PD-L1 treatment, suggesting that S100A9 can also function as an alarmin-like signal that supports early immune recruitment under certain conditions (Demir et al., 2024). Consistently, Luo et al. showed that perturbation of S100A8/A9 in the hematopoietic system or bone marrow could accelerate tumor progression by promoting protumorigenic myelopoiesis and attenuating T cell-mediated antitumor immunity (Luo et al., 2025). These findings argue against indiscriminate systemic suppression and indicate that tumor stage, immune contexture, treatment timing, and cellular source should be considered when designing S100A8/A9-targeted interventions.

Precision delivery may help improve the therapeutic index of S100A8/A9-directed therapy by restricting inhibition to pathological inflammatory niches while preserving beneficial immune-alert functions elsewhere. This rationale is supported by evidence that local release of S100A8/A9 inhibitors using a tumor-targeted drug-delivery system can exhibit antitumor activity while avoiding myelopoiesis-promoting effects (Luo et al., 2025). Nanomedicine provides one possible route, although direct S100A8/A9-focused evidence in CRC remains limited. In a C26 colon cancer model, DOTAP-containing nanoliposomes reduced MDSC population and activity in the spleen and decreased S100A8 and arginase-1 expression in splenic MDSCs (Taheri et al., 2022). This study supports the feasibility of modulating S100A8-associated myeloid suppression through delivery-system design, but it does not yet establish a tumor-targeted S100A8/A9 inhibitor platform. Future delivery strategies should therefore be evaluated for tissue specificity, bone-marrow effects, and compatibility with immune checkpoint blockade or receptor-blocking peptides.

Biologic engineering offers another selective strategy. Neutralizing single-chain variable fragment antibodies against S100A8 have recently been developed and shown to bind S100A8 and suppress S100A8-induced inflammatory signaling in macrophages and HT-29 colorectal cancer cells (Raeisi et al., 2025). This provides proof of concept for antibody-fragment-based S100A8 intervention, but the evidence remains *in vitro* and requires validation in CRC-relevant animal models. Together, these studies support a more refined therapeutic framework in which S100A8/A9 is targeted according to spatial distribution, disease stage, immune status, and combination-treatment context rather than through broad systemic inhibition.

## 5 Conclusion, limitations, and future prospects

This review has examined S100A8/A9 as a central inflammatory mediator in CRC, linking its engagement of TLR4 and RAGE and downstream signaling to a coordinated pro-tumorigenic program of inflammation, myeloid-driven immunosuppression, angiogenesis, and metastasis. The overarching message is that S100A8/A9 acts less as an isolated oncogenic factor than as a context-dependent hub coupling chronic inflammation to immune evasion in the CRC microenvironment, which also underlies its diagnostic and therapeutic relevance. The strength of evidence nonetheless varies across these processes: many biomarker findings remain correlative, and much of the mechanistic and therapeutic support is derived from preclinical models.

Several questions remain open. The cellular sources, spatial distribution, and temporal dynamics of S100A8/A9 across TME populations, including tumor cells, stroma, neutrophils, monocytes, macrophages, and MDSCs, are still poorly resolved, as are its interactions with other immunosuppressive pathways (e.g., STAT3 and TGF-β). S100A8 and S100A9 should not be treated as interchangeable, since their clinical associations and functional roles may differ by cellular compartment and molecular subtype, and the degree to which homologous S100 proteins compensate when S100A8/A9 is inhibited remains incompletely proven. Its role in therapy resistance, particularly resistance to ICIs in MSS CRC and in pre-metastatic niche formation, is also incompletely defined. Critically, S100A9 is not uniformly pro-tumorigenic: under some conditions it acts as an alarmin supporting early anti-tumor immunity, so indiscriminate blockade can be counterproductive. This context-dependence is a central caveat for any therapeutic strategy.

Therapeutically, S100A8/A9-directed approaches, including neutralizing antibodies, the small-molecule inhibitor tasquinimod, dual-receptor peptides, and natural compounds such as secoemestrin C, remain largely preclinical or derived from non-CRC settings. The most promising direction is rational combination rather than monotherapy: in preclinical CRC models, S100A8/A9-axis inhibition enhanced the activity of anti-PD-1 and anti-VEGF therapy (Bao et al., 2023; Deguchi et al., 2023), offering a potential route to improve immunotherapy responses in MSS CRC. Because broad systemic inhibition may itself be harmful in some immune contexts (Demir et al., 2024; Luo et al., 2025), clinical translation will depend on tissue-selective delivery to limit systemic toxicity and on biomarker-guided patient selection that identifies myeloid-enriched, immune-suppressed CRC subtypes.

In summary, S100A8/A9 is best viewed as a context-dependent biomarker and therapeutic node integrating inflammation, myeloid remodeling, metastasis, and treatment response in CRC. Its clinical value will depend on defining the right cellular source, disease stage, immune subtype, combination partner, and delivery strategy. Addressing these questions will require single-cell and spatial profiling, patient-derived organoids, and prospective, biomarker-stratified clinical evaluation. Validation of non-invasive S100A8/A9 measurement in serum, stool, or circulating tumor cells will also be essential for translating S100A8/A9 biology into precision-oriented CRC management.
